# *Enterococcus faecalis* and Endodontic Treatment Failure: A Qualitative Systematic Review of Its Role and Elimination Strategies

**DOI:** 10.1111/aej.70072

**Published:** 2026-03-16

**Authors:** Francine Santos Fernandes de Lima, Analia Gabriella Borges Ferraz Facury

**Affiliations:** ^1^ Centro Universitário Nossa Senhora Do Patrocínio School of Dentistry Itu São Paulo Brazil; ^2^ Department of Restorative Dentistry, School of Dentistry University of Uberaba Uberaba Minas Gerais Brazil

**Keywords:** *Enterococcus faecalis*, intracanal medication, root canal retreatment, systematic review, ultrasound

## Abstract

The aim of this study was to synthesize evidence on the influence of *Enterococcus faecalis* on endodontic treatment failure and strategies for its elimination. This study followed the Preferred Reporting Items for Systematic Reviews and Meta‐Analyses (PRISMA) guidelines and was registered in PROSPERO (CRD420251022741). PubMed and SciELO were searched using PICO terms, and 76 studies (1997 and 2025) met the inclusion criteria. Reported *E. faecalis* prevalence ranged from approximately 4%–78%, depending on detection methods and sampling strategies. Canal isolates frequently expressed virulence factors, formed biofilms, and survived alkaline pH and nutrient limitation, with multidrug resistance reported. Chemo‐mechanical preparation with sodium hypochlorite and EDTA, often combined with ultrasonic activation, reduced intraradicular bacterial load but achieved complete sterilization. In retreatment, two‐visit protocols with intracanal medication improved microbiological outcomes, although calcium hydroxide efficacy depended on vehicle and duration. Adjunctive approaches showed mainly in vitro promise. Optimised disinfection and sealing remain essential.

## 1 Introduction

Endodontic treatment aims to eliminate infection from the root canal system and promote the healing of periapical tissues. The success of endodontic treatment relies on effective microbial elimination from the root canal system and adequate sealing to prevent reinfection. Despite advances in techniques and materials, failures remain frequent, commonly manifesting as persistent apical periodontitis and requiring retreatment or surgical intervention [1, 2].

A major cause of these failures is the persistence of microorganisms in complex anatomical structures such as isthmuses, lateral canals, and dentinal tubules, which are not fully reached by chemomechanical preparation and irrigation. Within these niches, microbial biofilms enhance bacterial resistance to antimicrobial agents and host immune responses [3, 4].

Recent evidence confirms its epidemiological and clinical relevance, with *E. faecalis* being more commonly detected in secondary/persistent than in primary infections and even associated with salivary carriage that correlates with post‐treatment apical periodontitis [1, 5, 6].

Given these challenges, new therapeutic strategies have been investigated. Gold nanogel has shown antibacterial activity comparable to 2% chlorhexidine and superior to calcium hydroxide against *E. faecalis* in vitro [7]. Biodentine has demonstrated relevant antibacterial properties, often equivalent to or superior to other endodontic materials such as MTA [8]. Furthermore, quaternary ammonium compounds (QACs), such as K21 and benzalkonium chloride, have shown efficacy comparable to sodium hypochlorite, suggesting potential as alternative or adjunctive irrigants [9].

Understanding the prevalence, virulence, and resistance mechanisms of *E. faecalis* , along with evaluating novel therapeutic approaches, remains essential to improve clinical outcomes in endodontics. Therefore, the aim of this study was to collect data from the literature to better understand the influence of the presence of *E. faecalis* on endodontic treatment failure and to seek approaches for its elimination.

## 2 Materials and Methods

This systematic review was conducted in accordance with the Preferred Reporting Items for Systematic Reviews and Meta‐Analyses (PRISMA) guidelines and was registered in the PROSPERO international prospective register of systematic reviews (registration number: CRD420251022741). The review aimed to answer the following research question: “What is the role of *Enterococcus faecalis* in endodontic retreatment, and what are the most effective methods for its elimination during retreatment?”

The search strategy was developed based on the PICOS framework (Population, Intervention, Comparison, Outcome, and Study design) to ensure a comprehensive and focused literature search. Electronic searches were carried out in two databases: PubMed and SciELO. The search terms included Medical Subject Headings (MeSH) and keywords combined using Boolean operators (AND/OR), such as:

- PubMed: ((( *Enterococcus faecalis* ) OR (Streptococcus Group D) OR ( *Streptococcus faecalis* )) AND ((endodontic retreatment) OR (canal retreatment) OR (retreatment))) AND ((Root Canal Therapy) OR (Canal Therapies, Root) OR (Canal Therapy, Root) OR (Root Canal Therapies) OR (Therapies, Root Canal) OR (Therapy, Root Canal))
- SciELO: ( *Enterococcus faecalis* ) AND (endodontic)

The initial search was performed in October 2023 and subsequently updated in February 2025. Relevant studies were identified by screening titles and abstracts, and potentially eligible articles were retrieved for full‐text review. Data regarding study design, population, and main findings were extracted from each selected study. Articles that did not align with the primary focus of this review were reassessed to determine final eligibility based on predefined inclusion criteria.

### 2.1 Inclusion Criteria

- Original full‐text research articles published in English or Portuguese.
- Studies investigating the presence, persistence, virulence, or elimination of *Enterococcus faecalis* in the context of endodontic retreatment.
- Clinical studies involving patients with secondary or persistent endodontic infections.
- Laboratory studies (in vitro or ex vivo) using extracted human permanent teeth or root canal infection models contaminated with *E. faecalis* .
- Studies evaluating therapeutic interventions, materials, or protocols aimed at eliminating *E. faecalis* .

### 2.2 Exclusion Criteria

- Reviews, systematic reviews, or meta‐analyses.
- Case reports, expert opinions, or editorials.
- Studies on *E. faecalis* in primary endodontic infections.
- Studies involving deciduous or immature permanent teeth.
- Studies restricted to patients with systemic diseases.
- Studies primarily addressing prosthetic rehabilitation or intraradicular posts without evaluation of *E. faecalis* .
- Studies focused exclusively on infection model development without testing interventions against *E. faecalis* .
- Studies without relevant comparator or outcome related to *E. faecalis* detection, virulence, or elimination.

### 2.3 Critical Appraisal

According to the eligibility criteria and PRISMA guidelines, included articles were analysed independently by both reviewers. Any controversy between both reviewers was solved by a conversation between the two authors until agreement was achieved.

### 2.4 Data Extraction

Data from the full‐text articles were extracted independently by two reviewers. Initially, the selected studies were categorised into two main groups: (1) studies that investigated the performance and type of procedures used against *E. faecalis* (e.g., antimicrobial efficacy of irrigants, sealers, intracanal medications, or laser therapies) and (2) studies that identified or quantified the presence of *E. faecalis* in cases of endodontic retreatment and/or investigated its association with treatment failure.

The following data were extracted from each study:

- General study information (authors, year, country)
- Study design (in vitro, in vivo, or clinical trial)
- Sample characteristics (type of teeth, sample size)
- Microbiological evaluation methods (culture, PCR, etc.)
- Intervention or detection methods
- Main outcomes related to the presence, elimination, or impact of *E. faecalis* on retreatment prognosis

For studies assessing antimicrobial effectiveness, results were categorised based on the intervention tested and the degree of bacterial reduction achieved. For detection studies, the prevalence and clinical relevance of *E. faecalis* were summarised. All extracted data were compiled and organised according to the type of investigation and its relevance to the research question.

## 3 Results

**FIGURE 1:**
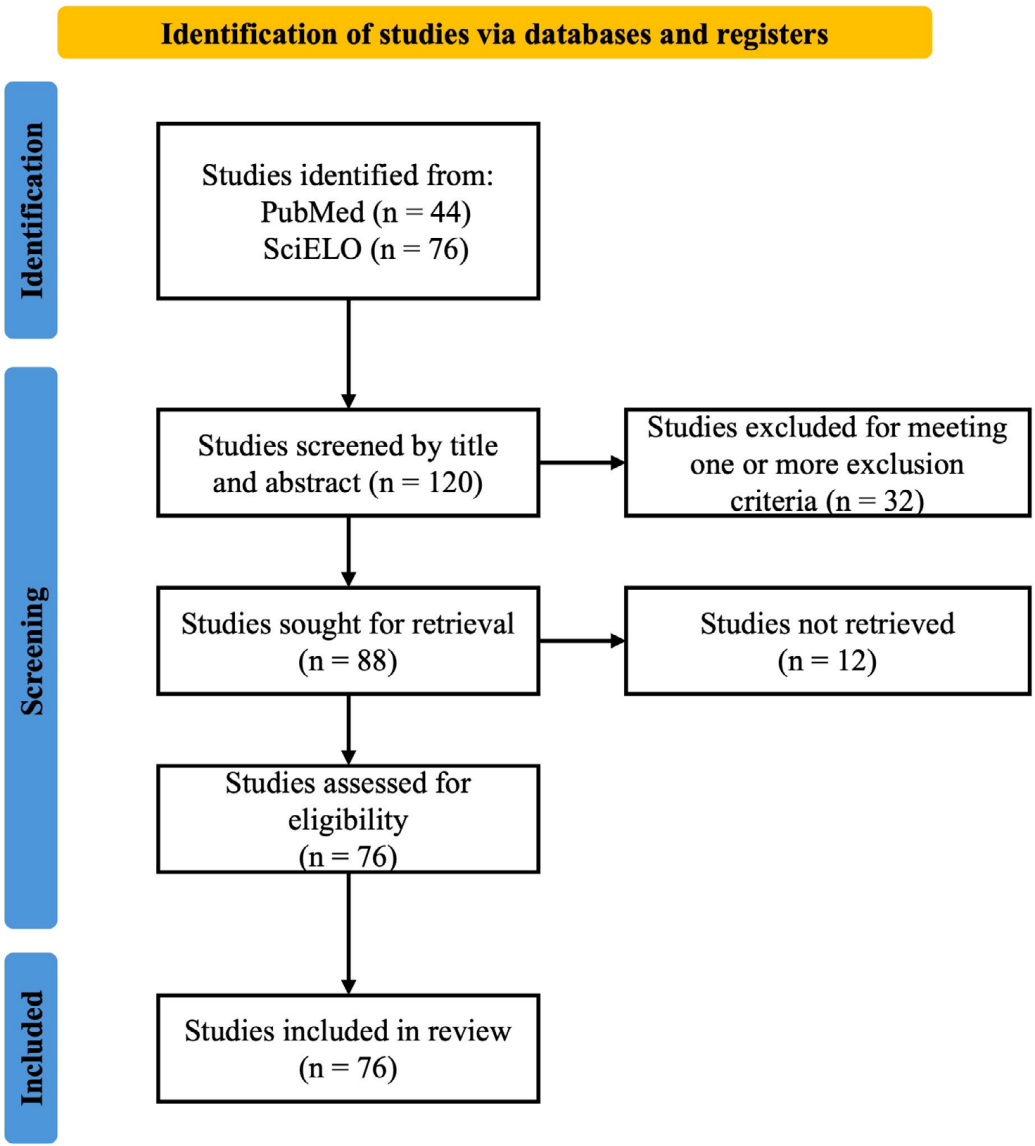
Flow chart of the search strategy.

The search strategy yielded 44 studies from PubMed and 76 studies from SciELO, totaling 120 studies. After analysing the titles and abstracts, 32 studies were excluded for not meeting the inclusion criteria. Following full‐text analysis, 76 studies met the eligibility criteria and were included in this systematic review. The included studies were published between 1997 and 2025. The flow chart of the search strategy for this systematic review has been summarised and is presented in Figure 1.

**FIGURE 2:**
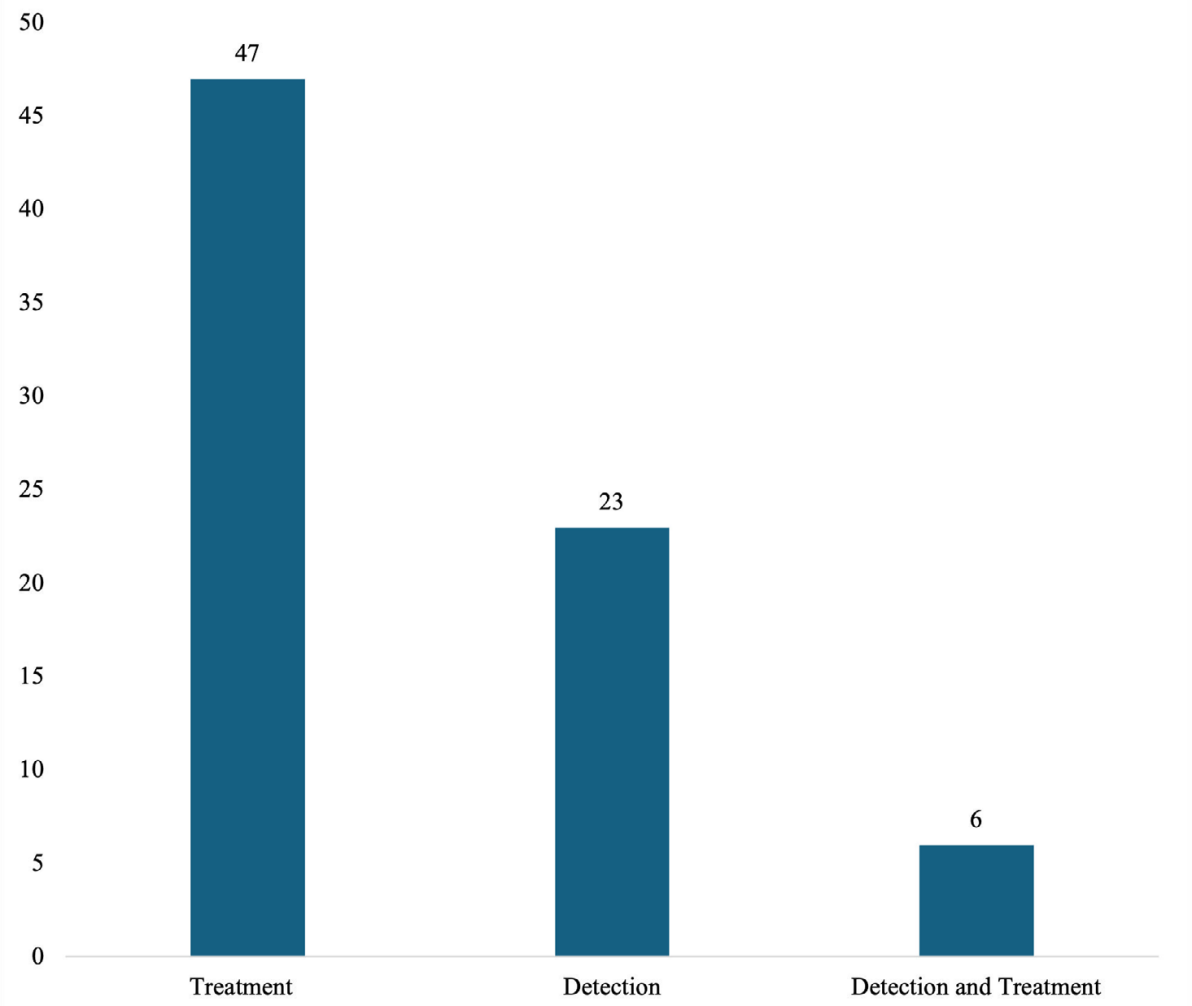
Distribution of included studies according to their primary focus.

To contextualise the scientific contributions of the included studies, they were categorised according to their primary focus related to *Enterococcus faecalis* (Figure 2). Of the 76 studies included, most investigated strategies aimed at reducing or eliminating *E. faecalis* from the root canal system. A smaller proportion focused on the detection, prevalence, or identification of the microorganism in endodontically treated teeth, while a limited number combined detection and treatment by evaluating microbial presence before and after therapeutic interventions. Detailed classifications of detection studies, combined detection and treatment studies, and overall study characteristics are presented in Tables 1, 2, 3, respectively.

**TABLE 1:** Classification of detection studies according to diagnostic methods, sample types, microbiological focus, and clinical context.

Study (author, year)	Culture	Molecular	Combined	RC	SAL	OTH	Prev	Vir	Res	Gen	Prim	Pers	Both
Al‐Sakati et al. [10] (2021)			X			X	X					X	
Angarita Díaz et al. [11] (2017)	X					X	X					X	
Barbosa‐Ribeiro et al. [12] (2016)	X			X			X	X				X	
Bronzato et al. [13] (2021)		X				X	X	X				X	
Gomes et al. [14] (2008)		X		X			X					X	
Hancock et al. [15] (2001)	X			X			X					X	
Lins et al. [16] (2024)		X		X					X	X	X		
Ozbek et al. [17] (2009)		X		X			X						X
Pandey et al. [18] (2016)	X			X			X					X	
Peciuliene et al. [19] (2000)	X			X			X					X	
Pinheiro et al. [20] (2003)	X			X			X					X	
Preethee et al. [21] (2012)		X		X				X				X	
Rôças et al. [22] (2008)		X		X			X					X	
Rôças et al. [23] (2012)		X		X			X					X	
Sakamoto et al. [24] (2008)		X		X						X		X	
Sánchez‐Sanhueza et al. [25] (2018)		X		X						X		X	
Sedgley et al. [26] (2006)			X	X	X		X	X					X
Seguel et al. [27] (2020)		X		X					X			X	
Siqueira et al. [28] (2004)		X		X			X					X	
Stevens et al. [29] (2009)	X			X								X	
Wang et al. [30] (2011)		X		X				X				X	
Wang et al. [31] (2012)			X	X	X		X						X
Zhu et al. [32] (2010)			X	X	X			X	X	X		X	

*Note:* An “X” indicates applicability.

Abbreviations: Both, primary and persistent infections; Combined, culture and molecular methods; Culture, culture‐based detection methods; Gen, genetic diversity/typing; Molecular, molecular detection methods; OTH, other sample types; Pers, persistent/secondary infection; Prev, prevalence/detection; Prim, primary infection; RC, root canal samples; Res, antimicrobial resistance; SAL, saliva/oral samples; Vir, virulence factors.

**TABLE 2:** Classification of combined detection and treatment studies according to diagnostic methods, treatment strategies, microbiological outcomes, and clinical context.

Study	Cult	Mol	Comb	Irrig	Ca(OH)_2_/high pH	Pro	Prev	Res	Gen	High‐pH	Prim	Pers	Both
Al‐Badah et al. [33] (2015)		X					X	X	X			X	
Schirrmeister et al. [34] (2007)			X	X	X		X					X	
Siren et al. [35] (1997)	X					X	X					X	
Sundqvist et al. [36] (1998)	X					X	X					X	
Weckwerth et al. [37] (2013)	X				X					X		X	
Williams et al. [38] (2006)			X				X						X

*Note:* An “X” indicates applicability.

Abbreviations: Both, primary and persistent infections; Ca(OH)_2_/High pH, calcium hydroxide–based medication or high‐pH conditions; Comb, combined culture and molecular methods; Cult, culture‐based detection; Gen, genetic diversity/typing; High‐pH, survival or resistance to alkaline conditions; Irrig, chemical irrigants; Mol, molecular detection; Pers, persistent/secondary infection; Prev, prevalence or detection before and/or after treatment; Prim, primary infection; Pro, procedural/clinical management factors; Res, antimicrobial resistance.

**TABLE 3:** Summary of the studies included in the systematic review.

Study	RR	Study type	Interventions tested	Main outcome
Al‐Badah et al. [33] (2015)	DT	Clinical observational	Analysis of *E. faecalis* from 82 root canal samples. Clonal diversity assessed via RFLP and RAPD‐PCR; antibiotic susceptibility tested for erythromycin, linezolid, and tetracycline.	*E. faecalis* was the most prevalent species. Five to six distinct genotypes were identified. Resistance varied: ~5% resistant to erythromycin, ~52% intermediate, and one isolate resistant to tetracycline and another intermediate to linezolid. Resistant strains tended to cluster by genotype.
Al‐Sakati et al. [10] (2021)	D	Clinical observational	Comparison of culture‐dependent vs. real‐time Pan‐PCR (16S rDNA for bacteria, ITS1‐2 for fungi) on gutta‐percha and plaque samples from reinfected, root‐filled teeth	All plaque samples (42/42) showed bacterial growth; gutta‐percha cultures were positive in 32/42 samples (mostly Streptococcus spp. and *E. faecalis*). Pan‐PCR detected microbial DNA in 31/42 gutta‐percha samples—including difficult‐to‐cultivate bacteria like Acinetobacter, Pyramidobacter, Synergistes, Atopobium, and Pseudoramibacter. Fungal DNA (Candida spp.) was detected in 5/42 root canals by PCR, despite negative cultures. This underscores that culture‐independent methods (Pan‐PCR) are more effective in detecting both culturable and non‐culturable microorganisms.
Alcalde et al. [39] (2017)	T	In vitro experimental	Investigated whether ultrasonic agitation of AH Plus sealer during obturation improves sealer penetration and antimicrobial effect against *E. faecalis*.	Ultrasonic agitation enhanced the intratubular antimicrobial activity and promoted better adaptation and distribution of the sealer.
Alves et al. [40] (2013)	T	In vitro experimental	Tested farnesol (0.2%), xylitol (5% and 20%), and their combination as endodontic irrigants. Assays included crystal violet (biofilm biomass), dentin disinfection (viability), and root canal disinfection models. Compared against saline and 2.5% NaOCl.	Farnesol best reduced biofilm biomass; xylitol affected biomass to a lesser extent. The farnesol/xylitol combo significantly outperformed saline in reducing bacterial load (*p* = 0.02), though NaOCl remained most effective across tests (*p* < 0.001).
Aminsobhani et al. [41] (2022)	T	Ex vivo experimental	Solvents (chloroform, orange oil, xylene, eucalyptol) used for gutta‐percha removal; antibacterial efficacy against *E. faecalis*	All solvents reduced bacterial load by > 99%; chloroform most effective, followed by orange oil and xylene (no difference), least effective: eucalyptol.
Amorim et al. [42] (2024)	T	In vitro experimental	Assessed the antimicrobial activity of chlorhexidine and camphorated paramonochlorophenol (CPMC) against oral pathogens including *E. faecalis*.	Both agents were effective, but CPMC showed broader and more potent antimicrobial effects. However, safety concerns with CPMC were acknowledged.
Angarita Díaz et al. [11] (2017)	D	Clinical observational	Analysis of cast metal cores used in clinical practice (*n* = 32); cores immersed in saline, serial dilutions plated on selective media for *E. faecalis*, S. aureus, and *C. albicans*	*E. faecalis* was detected in 1 core (3.2%), at ~5 × 10^4^ CFU/mL. S. aureus and *C. albicans* were absent. Other species found included Candida parapsilopsis (35.5%), C. tropicalis (6.5%), *Kocuria kristinae* (16.1%), *S. saprophyticus*(12.9%), and *Stenotrophomonas maltophilia* (3.2%).
Arias et al. [43] (2016)	T	In vitro experimental	Evaluated whether 1‐min ultrasonic agitation enhances the penetration and antimicrobial efficacy of calcium hydroxide paste in infected bovine dentin using confocal laser scanning microscopy (CLSM), CFU counts, and dye penetration.	Ultrasonic agitation significantly improved intratubular penetration and antimicrobial action against *E. faecalis*, especially in the middle and deeper third of the dentin.
Asnaashari et al. [44] (2017)	T	Clinical interventional	Photodynamic Therapy (LED + toluidine blue) vs Calcium Hydroxide Paste	Both reduced *E. faecalis*, but photodynamic therapy was significantly more effective.
Barbosa et al. [45] (2020)	T	In vitro experimental	Evaluated antibacterial activity of Bio‑C Sealer (calcium silicate‐based) against *E. faecalis*, *E. coli*, *P. aeruginosa*, *S. aureus*, and *S. mutans*; compared fresh vs. set sealers, using agar diffusion and biofilm (CFU) assays among multiple sealers.	Fresh Bio‑C Sealer demonstrated antibacterial activity against all tested bacteria except *S. mutans*. After setting, its activity against *E. faecalis* biofilm was comparable to other sealers (*p* > 0.05), but less effective than EndoFill against *S. mutans* biofilm (*p* < 0.05).
Barbosa‐Ribeiro et al. [12] (2016)	D	Clinical observational	20 single‐rooted teeth with post‐treatment apical periodontitis, randomized into two groups (*n* = 10 each): CMP with 2% CHX gel vs 6% NaOCl. Samples taken at three stages—S1 (before CMP), S2 (after CMP), S3 (after 30 days of ICM with Ca(OH)_2_ + 2% CHX gel). Cultivable bacteria counted (CFU); lipoteichoic acid (LTA) quantified via ELISA.	Cultivable bacteria reduced by ≥ 99.4% after CMP and ≥ 99.5% after ICM. LTA levels dropped by 24.8% after CMP and 38.6% after ICM (*p* < 0.05). CHX gel was significantly more effective than NaOCl in reducing both bacteria (99.3% vs 92.1%) and LTA (26.9% vs 22.6%) after CMP. ICM achieved 100% bacterial reduction with CHX group versus 98.5% with NaOCl; LTA reduction was similar in both groups (43.2% vs 36.2%, *p* > 0.05). *E. faecalis* remained the most frequently isolated taxon across all stages.
Bennie et al. [46] (2016)	T	In vitro experimental	Compared different irrigants (e.g., NaOCl, EDTA, chlorhexidine, MTAD) for their smear layer removal and antibacterial activity against *E. faecalis*.	EDTA and MTAD were most effective at smear layer removal, while NaOCl had the strongest antibacterial effect. Combining irrigants was recommended for optimal results.
Brar et al. [47] (2019)	T	In vitro experimental	This study evaluated the antibacterial activity of Triphala (a traditional Ayurvedic herbal formulation) and turmeric extract against *Enterococcus faecalis* biofilms.	Both herbal agents showed antibacterial activity, with Triphala slightly outperforming turmeric. However, both were less effective than sodium hypochlorite. These results support further investigation of plant‐based irrigants for endodontic use.
Bronzato et al. [13] (2021)	D	Clinical observational	Microbial profile of periapical lesions via PCR; quantification of LPS and LTA levels	Detected bacterial DNA, LPS, and LTA in all samples; *P. micra* most prevalent followed by *E. faecalis*; no association with treatment type; LPS and LTA correlated with clinical features, but no link with specific bacteria.
Bruniera et al. [48] (2020)	T	In vitro experimental	Developed a green‐synthesized silver nanoparticle (AgNP)‐based hydrogel dressing. Evaluated MIC and MBC against *E. faecalis*, *E. coli*, *P. aeruginosa*, *S. aureus*, and *S. mutans* using NCCLS protocols.	The AgNP formulation was well‐characterized and demonstrated antimicrobial activity across all tested bacterial strains, indicating its potential as an intracanal medicament.
Castilho et al. [49] (2013)	T	In vitro experimental	Compared the antimicrobial activity of Amazonian plant extracts to 2% chlorhexidine against *E. faecalis* using agar diffusion and broth microdilution methods.	Some extracts showed significant antibacterial effects, though chlorhexidine was still more effective. Results indicate potential for plant‐based alternatives in endodontic disinfection.
Castilho et al. [50] (2014)	T	In vitro experimental	Screened Amazonian plant extracts (by microdilution and disk diffusion) for activity against *E. faecalis* (ATCC 29212); phytochemical profile analyzed via TLC and HPLC.	Extracts from *Ipomoea alba*, Diclinanona calycina, and Moronobea coccinea exhibited strong bactericidal activity (MIC ≤ 80 μg/mL); several additional extracts showed significant inhibition zones. These extracts are promising candidates for anti‐*E. faecalis* agents.
Choi et al. [51] (2018)	T	In vitro experimental	Investigated the efficacy of N‐acetylcysteine (NAC) in disrupting and eliminating multispecies endodontic biofilms including *E. faecalis*, *F. nucleatum*, and *P. intermedia*.	NAC was effective in biofilm removal and bacterial killing, showing potential as an endodontic irrigant alternative or adjuvant.
Costa et al. [52] (2010)	T	In vitro experimental	Evaluated the antimicrobial activity and cleaning efficacy of hydroalcoholic plant extracts (*Schinus terebinthifolius*, Astronium urundeuva, Syderoxylum obtusifolium, *Ximenia americana*) compared with 2.5% NaOCl and 0.12% CHX against *E. faecalis*. Also assessed smear layer removal by SEM.	All extracts demonstrated antimicrobial activity against *E. faecalis*. The quixabeira extract achieved the most effective cleaning in the apical third—though no method completely removed the smear layer
Costa et al. [53] (2012)	T	In vitro experimental	Assessed the cleaning efficiency and antimicrobial activity of plant extracts (including Piper regnellii and Amburana cearensis) against root canal pathogens using microbiological tests and SEM imaging.	Some extracts showed effective antimicrobial action against *E. faecalis* and improved smear layer removal, suggesting potential as auxiliary irrigants in endodontics.
Souza Borges et al. [54] (2021)	T	In vitro experimental	Tested cinnamaldehyde and α‐terpineol (10 mg/mL) against mono‐ and dual‐species biofilms of *C. albicans* and *E. faecalis*; assessed metabolic activity, viability (CFU), and phospholipase activity.	Both compounds significantly reduced metabolic activity, viability, and enzyme activity in mono‐species biofilms (*p* < 0.05); α‐terpineol also effective against dual‐species biofilms (*p* < 0.05). Activity was comparable to 2.5% NaOCl and 2% CHX.
Santi et al. [55] (2015)	T	Ex vivo experimental	Assessed susceptibility of facultative anaerobic bacteria (*E. faecalis*, *E. faecium*, *A. viscosus*, *S. aureus*) isolated from persistent endodontic infections to systemic antibiotics.	Specific susceptibility data not retrievable; citation confirmed study focus.
Duque et al. [56] (2023)	T	In vitro experimental	This study investigated the synergistic antibacterial effect of epigallocatechin‐3‐gallate (EGCG), a green tea polyphenol, and fosfomycin against multispecies biofilms associated with endodontic infections, particularly *E. faecalis*.	The combination of EGCG and fosfomycin significantly reduced biofilm biomass and bacterial viability compared to either agent alone, suggesting potential for adjunctive use in root canal disinfection protocols.
Duque‐Aristizabal et al. [57] (2019)	T	In vitro experimental	Antibacterial capacity of silver nanoparticles (AgNPs) integrated into zinc oxide–eugenol cement.	AgNPs were successfully incorporated into ZOE cement and demonstrated significant bactericidal activity against *Enterococcus faecalis*, evidenced by inhibition zones in agar diffusion assays.
Edgar et al. [58] (2006)	T	In vitro experimental	Gutta‑percha removal using chloroform vs saline in human teeth inoculated with *E. faecalis*	Chloroform significantly reduced cultivable *E. faecalis*. Mean CFU after chloroform: 21 ± 56 CFU/mL vs 280 ± 480 with saline. Negative cultures in 11/17 chloroform samples vs none with saline. After apical enlargement, 9/17 negative in chloroform vs 1/17 with saline (*p* < 0.05).
Estrela et al. [59] (2012)	T	In vitro experimental	Human maxillary anterior teeth infected with *E. faecalis* for 60 days; evaluation of antimicrobials applied via positive‐ or negative‐pressure irrigation	Cetylpyridinium chloride (CPC) at 0.1% and 0.2% significantly reduced *E. faecalis*. In agar diffusion tests, CPC produced antimicrobial zones comparable to 2% chlorhexidine and larger than 2.5% NaOCl, demonstrating its substantial antibacterial potential.
Galoza et al. [60] (2015)	T	In vitro experimental	Assessed the effect of dentin powder on pH and antimicrobial activity against *E. faecalis* in various calcium hydroxide formulations—associated with propylene glycol (G1), camphorated paramonochlorophenol and glycerin (G2), or 2% chlorhexidine gluconate (G3)—evaluated at 24 h, 7, 14, and 21 days.	Dentin had no significant effect on pH or antimicrobial activity, except a transient pH reduction in the propylene glycol formulation at 24 h. Antimicrobial efficacy (descending): G2 ≈ G2 + dentin > G3 ≈ G3 + dentin > G1 ≈ G1 + dentin.
Gomes et al. [14] (2008)	D	Clinical observational	PCR detection of nine bacterial taxa in root‐filled teeth with periapical lesions	*Enterococcus faecalis* was most prevalent (77.8%), followed by *Peptostreptococcus micros* (51.1%); several species were significantly associated with clinical signs such as percussion tenderness, pain, abscesses, and purulent exudate.
Guerreiro‐Tanomaru et al. [61] (2011)	T	In vitro experimental	2.5% NaOCl, 2% CHX, and 1% peracetic acid via direct contact at variable exposure times.	Both NaOCl and CHX eradicated *E. faecalis* within 30 seconds. Peracetic acid achieved an 86% bacterial reduction in 3 min and complete elimination by 10 min — effective but slower.
Hancock et al. [15] (2001)	D	Clinical observational	Culture‐based analysis of root canal samples from 54 teeth with failed root canal treatment	Bacteria were isolated in 34 of 54 teeth; *Enterococcus faecalis* was the most common species, present in approximately 30% of positive cultures.
Kuga et al. [62] (2013)	T	In vitro experimental	Assessed pH, calcium ion release, and antimicrobial activity (agar diffusion) of MTA Fillapex vs. Sealapex and AH Plus over 24 h, 14, and 28 days.	Sealapex showed the highest pH across all times; MTA Fillapex had intermediate pH and calcium release compared to Sealapex and AH Plus. Antimicrobial activity against *E. faecalis* was similar across all sealers; Sealapex was more effective against S. aureus.
Lins et al. [16] (2024)	D	Clinical observational	MLST genetic analysis of *E. faecalis* isolates collected between 2010 and 2023 from primary endodontic infections in Southeast Brazil.	Identified 12 sequence types (STs); ST 397 was highly prevalent (15/32). Resistance was highest for clindamycin (100%), tetracycline (34.4%), azithromycin (31.2%), and ciprofloxacin (19.2%). One isolate was multidrug‐resistant. Indicates a dominant, resistant clone circulating over a 10‐year period in the region.
Machado et al. [63] (2011)	T	In vitro experimental	Evaluated calcium hydroxide vs. iodoform in culture broth against *E. faecalis*, at 0, 7, 14, and 21 days; morphology assessed by transmission electron microscopy on day 7.	Both agents reduced bacterial growth between days 7 and 14, achieving elimination by days 14–21. TEM revealed irreversible ultrastructural damage to *E. faecalis* cells.
Massunari et al. [64] (2017)	T	In vitro experimental	Tested aqueous (PCAE) and hydroethanolic (PCHE) extracts of *Psidium cattleianum* leaves against *E. faecalis*, *P. aeruginosa*, A. israelii, and *C. albicans* (MIC, MLC, biofilm assays); biocompatibility assessed in rats via subcutaneous implant histology.	MIC and MLC ranged 0.25–4 mg/mL. PCHE eliminated all tested strains (except *C. albicans*); PCAE eliminated *E. faecalis* and P. aeruginosa. Both extracts eradicated *E. faecalis* biofilms; only PCHE eliminated P. aeruginosa biofilms. Both were biocompatible; PCHE induced modest, declining inflammation.
Mozini et al. [65] (2009)	T	In vitro experimental	Evaluated how different lengths of remaining root canal filling (2 mm, 4 mm, and 6 mm) after post space preparation influenced *E. faecalis* coronal leakage.	Greater leakage occurred when only 2 mm of filling remained. Increasing the length of remaining gutta‐percha significantly reduced bacterial penetration.
Mukorera et al. [66] (2022)	T	In vitro experimental	Tested antimicrobial effects of Sealapex, EndoREZ, and GuttaFlow Bioseal against *E. faecalis* in both fresh and aged conditions over 7, 14, 21, and 28 days.	All three sealers demonstrated antibacterial activity across the testing periods, suggesting effectiveness in entombing or reducing residual *E. faecalis* in root canals.
Müller et al. [67] (2025)	T	In vitro experimental	Evaluated fresh (unsetting) calcium silicate–based (Bio‑C Sealer, Sealer Plus BC) and epoxy resin–based (AH Plus Jet) sealers for antibacterial, antibiofilm, and cell viability effects against *E. faecalis*, using agar diffusion, confocal microscopy, viability assays, and pH measurements.	All sealers inhibited *E. faecalis* growth. Calcium silicate–based sealers (Bio‑C Sealer and Sealer Plus BC) showed effective antibiofilm activity and influenced cell viability, with pH‐related mechanisms noted.
Ozbek et al. [17] (2009)	D	Clinical observational	Real‐time SYBR Green PCR detection of *Enterococcus faecalis* in 79 root canal samples: 43 failed endodontic treatments vs. 36 primary infections.	*E. faecalis* detected in 74.4% of failed retreatment cases versus 25% of primary infections (p < 0.01), indicating a significantly stronger association with treatment failures. Overall prevalence was 51.9%.
Pandey et al. [18] (2016)	D	Clinical observational	Correlation between clinical symptoms and root canal pathogens	Positive correlation between tenderness to percussion and *E. faecalis*; also between pain and *S. mitis*.
Peciuliene et al. [19] (2000)	D	Clinical observational	Standard retreatment procedures with NaOCl + EDTA irrigation.	*E. faecalis* was the dominant species in retreatment cases of apical periodontitis.
Pelozo et al. [68] (2023)	T	Clinical interventional	Root canal retreatment (RCR) + 980‑nm diode laser vs placebo (RCR only)	Laser provided significant microbial reduction pre‑prep (42% total, 53% *E. faecalis*) and 45% more healed casesat 12 months follow‑up.
Pinheiro et al. [20] (2003)	D	Clinical observational	Culture‐based microbial analysis of previously root‐filled teeth with persistent periapical lesions	Microorganisms were isolated from 51 of 60 teeth, mostly involving one or two strains per canal. Facultative anaerobes and Gram‐positive bacteria predominated, with *E. faecalis* as the most frequent species. Some clinical signs were significantly associated with specific microbes, such as Peptostreptococcus with symptoms, and Streptococcus or Candidawith coronal leakage.
Pinheiro et al. [69] (2009)	T	In vitro experimental	Evaluated the antimicrobial action of three endodontic sealers (Acroseal, Polifil, and Epiphany) against *E. faecalis* using the agar diffusion method.	Acroseal and Polifil exhibited higher antimicrobial activity than Epiphany. Epiphany showed significantly smaller inhibition zones, indicating lower effectiveness against *E. faecalis*.
Preethee et al. [21] (2012)	D	Clinical observational	PCR detection of the efaA virulence gene (endocarditis antigen) in *E. faecalis* isolates from therapy‐resistant root canals	The efaA gene was found in 11 out of 15 *E. faecalis*‐positive samples (~73%), indicating that this virulence factor is present in resistant endodontic infections similarly to "medical" strains.
Ramírez‐González et al. [70] (2025)	T	In vitro experimental	Evaluated endodontic files coated with silver nanoparticles for antimicrobial performance against *E. faecalis* and biofilm formation.	The nanoparticle‐coated files exhibited superior antimicrobial activity compared to uncoated files, suggesting enhanced clinical potential for infection control.
Rendón Gómez et al. [71] (2017)	T	In vitro experimental	Evaluated coronal microleakage using *E. faecalis* penetration in teeth filled by warm vertical compaction, lateral compaction, and single‐cone techniques.	Differences in leakage patterns were observed among techniques, with specific methods showing better resistance to *E. faecalis* penetration.
Rezende et al. [72] (2016)	T	In vitro experimental	Compared antimicrobial activity of calcium hydroxide–based sealer (Sealapex), resin‐based sealer (AH Plus), and Acroseal against *E. faecalis* biofilm over different settings times using agar diffusion/direct contact.	Sealapex exhibited the strongest antimicrobial action across all time points. Acroseal and AH Plus showed delayed activity (only after 14 days). None completely eliminated the biofilm.
Rôças et al. [22] (2008)	D	Clinical observational	Detection of 28 bacterial taxa via reverse‐capture checkerboard hybridization and species‐specific PCR for *E. faecalis* and *C. albicans* in treated root canals	Bacterial DNA detected in all samples. Common taxa included Streptococcus (47%), Lactobacillus (35%), T. denticola, *P. intermedia*, among others (29%). *E. faecalis* was found in 47% and *C. albicans* in 6%. Persistent infections were generally mixed, and *E. faecalis* was not the most dominant species when present.
Rôças et al. [23] (2012)	D	Clinical observational	PCR detection of 28 taxa including *E. faecalis* in treated‐root canal teeth	Propionibacterium spp. most prevalent, followed by *Fusobacterium nucleatum*, Streptococci, *P. alactolyticus*; *E. faecalis*present in 38% of cases (9.8% of total bacteria).
Rodrigues et al. [73] (2015)	T	Clinical interventional	Compared two instrumentation systems: Self‑Adjusting File (SAF) vs. Twisted File Adaptive (TFA), with passive ultrasonic irrigation (PUI) adjunct in TFA group.	Both systems significantly reduced intracanal bacterial load (*p* < 0.001).
Sakamoto et al. [24] (2008)	D	Clinical observational	16S rRNA gene clone library analysis of root canal microbiota in failed endodontic cases	Bacteria detected in all cases; 74 distinct taxa across six phyla identified, including many uncultivated phylotypes (55%). Mixed infections with mean of 10 taxa per tooth; high inter‐individual variability. Indicates presence of novel candidate pathogens beyond *E. faecalis*.
Sánchez‐Sanhueza et al. [25] (2018)	D	Clinical observational	Used molecular typing (PFGE and MLST) to investigate the genetic diversity of *E. faecalis* strains isolated from root canals of teeth with persistent endodontic infections.	Found clonal diversity among isolates; certain genotypes were more associated with persistent infections, suggesting adaptive traits aiding survival in root canals.
Schirrmeister et al. [34] (2007)	DT	Clinical interventional	Culture & PCR detection in asymptomatic root‐filled teeth; NaOCl + EDTA irrigation and Ca(OH)_2_ dressing applied sequentially	Culture positive in 60%, PCR in 65%; *E. faecalis* DNA found in 31% samples; after NaOCl + EDTA, no microorganisms detected; CHX and Ca(OH)_2_ added no further benefit.
Sedgley et al. [26] (2006)	D	Clinical observational	Culture and PCR for detection; also assessed virulence traits.	*E. faecalis* detected in 68% of patients in at least one oral site, but only 5% in root canal samples. PCR showed significantly higher sensitivity than culture (32% vs. 4%).
Seguel et al. [27] (2020)	D	Clinical observational	Evaluated antibiotic resistance phenotypes and genotypes of *E. faecalis* isolates from persistent endodontic infections. Assessed susceptibility, presence of ermB gene, and resistance profiles.	Isolates displayed multi‐drug resistance phenotypes, notably complete resistance to metronidazole; highest MICs for tetracycline and erythromycin; all were susceptible to amoxicillin/clavulanic acid. Some isolates harbored the ermB gene conferring erythromycin resistance; phenotypic–genotypic correlation was not significant (*p* > 0.05).
Silva et al. [74] (2014)	T	In vitro experimental	Evaluated antimicrobial effects of photodynamic therapy on *E. faecalis* using methylene blue (MB) and malachite green (MG) as photosensitizers, irradiated by diode laser for 30–120 seconds.	Significant reductions in *E. faecalis* viability were observed with both MB and MG at 60 and 120 seconds. Indicates PDT with these photosensitizers may be effective as an adjunct in endodontic disinfection
Silva et al. [75] (2014)	T	In vitro experimental	Compared EndoBinder (a calcium aluminate–based sealer) to white MTA, examining cytotoxicity, antimicrobial activity, pH, solubility, and water sorption.	Under study conditions, EndoBinder showed favorable biological and physicochemical properties comparable to MTA—suggesting it may be suitable for applications like root resorption treatments, perforation repairs, and root‐end fillings
Silva et al. [76] (2021)	T	In vitro experimental	Evaluated a digital electrofulguration device (Endox Endodontic System, EES) for eliminating *E. faecalis* in contaminated root canals. Compared CHX gel, NaOCl, saline—with or without EES treatment—at multiple time points.	EES alone did not significantly reduce bacterial counts. After standard chemo‐mechanical preparation (CMP), significant CFU reduction occurred equally with CHX and NaOCl. EES after CMP provided no additional benefit, suggesting current endodontic disinfectants remain more reliable than electrofulguration alone.
Silveira et al. [77] (2007)	T	In vivo experimental (animal)	Assessed periapical tissue repair following root canal treatments done in one or two visits, using calcium hydroxide or camphorated paramonochlorophenol as intracanal medications.	Two‐visit treatments with medication showed superior periradicular healing compared to single‐visit treatment, supporting the role of intracanal medication in enhancing repair.
Siqueira et al. [28] (2004)	D	Clinical observational	PCR screening of 19 microbial taxa in retreatment cases	Microorganisms were identified in all samples; *E. faecalis* was most prevalent (77%), followed by *Pseudoramibacter alactolyticus* (52%), *Propionibacterium propionicum* (52%), *Dialister pneumosintes* (48%), and *Filifactor alocis* (48%). Candida albicans was found in 9%. Teeth with root fillings extending more than 2 mm from the radiographic apex had a significantly higher species count per sample (mean 5) compared to those with shorter fillings (mean 3) (*p* < 0.05)
Siren et al. [35] (1997)	DT	Clinical observational	Cultures from root canal‐treated cases categorized as "enteric bacteria" (including *E. faecalis*) vs. "non‐enteric"; dentist treatment protocols assessed via questionnaire	Enteric bacteria were found significantly more often in canals that had been left unsealed between appointments (55% vs. 30%); they also correlated with a higher number of appointments and retreatment cases. Highlights the importance of maintaining aseptic and sealed environment during endodontic treatment.
Soligo et al. [78] (2018)	T	In vitro experimental	Grape seed extract (50%), calcium hypochlorite (6%), and sodium hypochlorite (6%) combined with rotary or reciprocating instrumentation in *E. faecalis*‐infected canals.	All active solutions showed similar efficacy in reducing bacterial counts, outperforming the saline control. The type of instrumentation (ProTaper Next vs Reciproc R25) did not affect the outcome.
Stevens et al. [29] (2009)	D	In vitro experimental	Induction and characterization of bacteriophages in *E. faecalis* isolates via mitomycin C induction	Induction and characterization of bacteriophages in *E. faecalis* isolates via mitomycin C induction
Sundqvist et al. [36] (1998)	DT	Clinical interventional	Microbial analysis of previously root‑filled teeth with periapical lesions; followed after conservative retreatment	The microbial flora was predominantly single‐species, gram‐positive; *E. faecalis* most commonly recovered. Conservative endodontic retreatment achieved a 74% success rate, influenced negatively by baseline infection and lesion size.
Vasconcelos et al. [79] (2017)	T	In vitro experimental	Assessed the impact of ultrasound streaming (US) on bacterial disinfection of flattened root canals prepared with ProTaper Universal, BioRaCe, and Reciproc systems; irrigation used saline, evaluated via CFU counts.	US significantly enhanced bacterial reduction even with saline alone. ProTaper Universal achieved complete elimination (0 CFU/mL) regardless of US. BioRaCe with US also reached 0 CFU/mL but only 30 without US. Reciproc performed less effectively (2.5 vs 5 CFU/mL with and without US). This suggests both the instrumentation system and US irrigation method influence disinfection success.
Vianna et al. [80] (2005)	T	In vitro experimental	Tested calcium hydroxide pastes prepared with various vehicles (saline, anesthetic solution, polyethylene glycol) against *E. faecalis* and Candida albicans.	Calcium hydroxide with polyethylene glycol showed better antimicrobial action, especially against *C. albicans*. *E. faecalis* was more resistant overall, confirming its relevance in persistent infections.
Wainstein et al. [81] (2016)	T	In vitro experimental	Compared GuttaFlow 2 (silicone‐based with modified silver particles), AH Plus (epoxy resin–based), and Endofill (zinc oxide–eugenol–based) against *E. faecalis* (ATCC 29212) using agar diffusion and direct contact tests (post‐setting).	GuttaFlow 2 showed no antibacterial effects. Inhibition zones were similar for Endofill and AH Plus; only Endofill reduced bacterial growth in the direct contact test. Thus, the silver modification in GuttaFlow 2 failed to confer antimicrobial benefits.
Wang et al. [30] (2011)	D	Clinical observational	*E. faecalis* isolates from retreatment cases (detection rate 39.3%); biofilm formation assessed via microplate reader and SEM; gelE expression measured by real‐time qPCR	Most isolates formed biofilm; strains from teeth without sinus tract showed stronger biofilm formation. gelE expressionwas significantly higher in cases with apical radiolucency and in biofilm‐positive strains.
Wang et al. [31] (2012)	D	Clinical observational	Culture on selective media and 16S rRNA identification.	Prevalence of *E. faecalis* was 19% in saliva and 38% in root canals. Presence in saliva increased odds of canal colonization (OR = 9.7). Unsatisfactory obturation associated with higher bacterial counts.
Weckwerth et al. [37] (2013)	DT	In vitro experimental	Evaluated the resistance of *E. faecalis* (20 clinical isolates + ATCC 29212) to alkaline pH levels (9.5, 10.5, 11.5, 12.5) over periods up to 14 days. Turbidity assessed against McFarland standards.	Evaluated the resistance of *E. faecalis* (20 clinical isolates + ATCC 29212) to alkaline pH levels (9.5, 10.5, 11.5, 12.5) over periods up to 14 days. Turbidity assessed against McFarland standards.
Williams et al. [38] (2006)	DT	Clinical observational	qPCR, RT‐PCR vs cultivation for *E. faecalis* detection in endodontic treatment stages	qPCR detected significantly more *E. faecalis* than culture (*p* < 0.001); RT‐PCR revealed viable but non‐culturable bacteria missed by cultivation.
Zancan et al. [82] (2018)	T	In vitro experimental	Compared resistance of lab strain ATCC 29212 (urinary origin) vs. clinical canal strain ATCC 4083 against various intracanal medicaments (e.g., CH/S, CH/CHX, CMCP combinations, DAP, TAP).	The urinary‐derived strain was more resistant overall. Drug susceptibility rankings differed between strains. Authors recommend using ATCC 4083 for in vitro research to better reflect clinical behavior.
Zandi et al. [83] (2016)	T	Clinical interventional	Irrigation with 1% NaOCl vs 2% CHX, plus Ca(OH)_2_ medication	Both irrigants decreased bacterial counts and number of infected canals; no significant difference between them.
Zerella et al. [84] (2005)	T	Clinical interventional	Ca(OH)_2_ in water vs Ca(OH)_2_ + 2% CHX slurry as interappointment medication during retreatment	Both medications disinfected failed root‐filled teeth effectively; no significant difference (60% vs 80% success, *p* > 0.05).
Zhu et al. [32] (2010)	D	Clinical observational	Collection of saliva and root canal samples in retreatment cases; phenotypic tests, antibiotic susceptibility, virulence gene detection, and PFGE typing of *E. faecalis* isolates	Prevalence of *E. faecalis* was 18.8% in saliva and 40.6% in root canals (*p* = 0.666); 19 isolates identified (6 from saliva, 13 from canals). All isolates carried virulence genes ace, asa, gelE, cylA, and efaA; PFGE revealed 62–100% genetic similarity. Indicates potential virulence in both oral and canal strains and that individual patients may harbor different strains.
Zordan‐Bronzel et al. [85] (2021)	T	In vitro experimental	Evaluated a new premixed calcium silicate‐based sealer regarding pH, setting time, solubility, cytocompatibility, and antibiofilm activity against *E. faecalis*.	The new sealer demonstrated alkaline pH, acceptable physicochemical properties, good cytocompatibility, and antibiofilm efficacy, indicating potential for clinical use.

Abbreviations: D, detection; DT, detection and treatment; RR, Role in the review; T, treatment.

**FIGURE 3:**
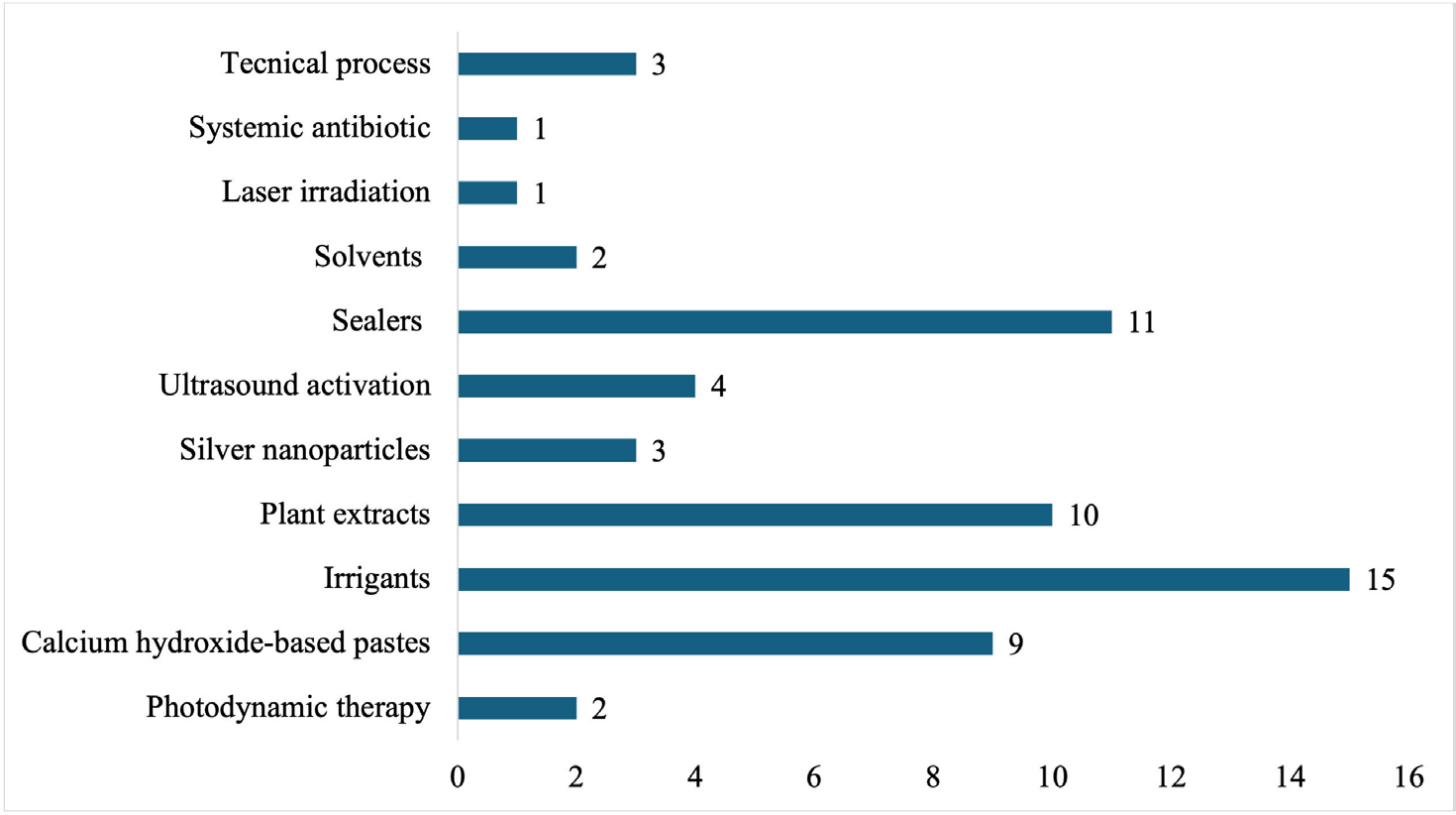
Distribution of treatment strategies evaluated against *Enterococcus faecalis* .

Among studies addressing treatment strategies (Figure 3), a wide range of approaches was evaluated, including photodynamic therapy, calcium hydroxide–based pastes, chemical irrigants (such as sodium hypochlorite, chlorhexidine, and peracetic acid), plant extracts, silver nanoparticles, ultrasound activation, laser irradiation, solvents, and different types of endodontic sealers. These investigations comprised mainly in vitro studies, as well as ex vivo and clinical approaches involving extracted teeth or samples from patients with persistent apical periodontitis. Antimicrobial effectiveness was assessed using culture‐based methods, molecular techniques such as quantitative PCR, and microbial viability or biofilm assays.

Sodium hypochlorite (NaOCl) was the most commonly used irrigant, frequently associated with EDTA, chlorhexidine, or ultrasonic agitation. Calcium hydroxide was tested in different vehicles, and some studies used experimental sealers or herbal compounds.

In studies related to detection, the prevalence of *E. faecalis* ranged from 5% to 77%, particularly in root‐filled teeth requiring retreatment. Molecular methods such as PCR showed higher sensitivity compared to culture. Root canal samples were the most commonly analysed specimens. Virulence genes like gelE, efaA, and cylA were identified in multiple isolates, and antibiotic resistance was frequently reported, notably to erythromycin, tetracycline, and ciprofloxacin. Genetic analyses revealed both clonal diversity and persistence of dominant sequence types, such as ST 40 and ST 397.

In combined detection and treatment studies, *E. faecalis* was identified before and after disinfection procedures, showing reduction after irrigation and medication. Sodium hypochlorite (NaOCl) and EDTA were widely used, with studies showing a decrease in microbial load, although detection of *E. faecalis* still occurred after treatment in some cases. Molecular methods confirmed higher detection rates compared to culture. The ability of *E. faecalis* to resist high pH environments and survive prolonged exposure to disinfectants was also reported.

**FIGURE 4:**
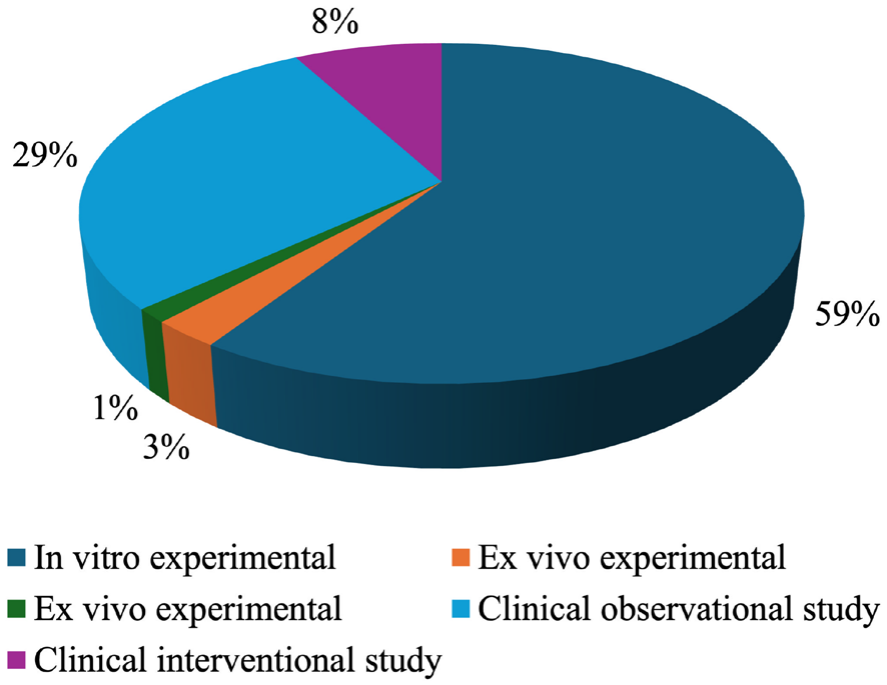
Distribution of included studies according to study design.

Most studies were conducted in vitro (Figure 4), and a high number originated from Brazilian research groups. The majority were published between 2010 and 2024, with the earliest study dating from 1997 and the most recent from 2025.

## 4 Discussion

Across the 76 studies, reported prevalence of *E. faecalis* varied widely, chiefly due to methodological and sampling heterogeneity. Culture tended to yield lower detection than species‐directed PCR in failed cases, whereas broad 16S clone‐library surveys often portrayed *E. faecalis* as infrequent within polymicrobial communities—an assay‐driven disparity that reconciles discordant signals [14, 15, 19, 24, 28]. Site and timing also mattered: *E. faecalis* was common in saliva/tongue and variably present in canals, with saliva positivity correlating with canal detection in retreatment; periapical tissues typically harboured anaerobic consortia in which *E. faecalis* was not dominant, and removed cast posts carried *E. faecalis*, Candida and Staphylococcus, highlighting reservoir‐mediated reseeding [11, 13, 26, 31]. Because many molecular studies lacked viability controls, residual DNA may inflate positivity; when biochemical or quantitative markers were tracked, LTA/LPS patterns supported ongoing microbial activity in a subset [12, 13]. These patterns are broadly in line with the Alghamdi and Shakir [86] systematic review, which, based on 11 studies, also reported a wide prevalence range and showed that *E. faecalis* may be highly prevalent in some retreatment series (often > 70%) but detected in much lower proportions in others. That review emphasised that earlier culture‐ and PCR‐based investigations frequently identified *E. faecalis* as the leading pathogen in failed root‐filled teeth, whereas more recent studies increasingly report other taxa—such as *Parvimonas micra* , *Solobacterium moorei* and *Fusobacterium nucleatum* —as predominant, with *E. faecalis* being a recurrent but not invariably dominant species. By synthesising 76 studies spanning both older and newer methodologies, the present review confirms the consistent association of *E. faecalis* with post‐treatment disease but supports a more polymicrobial view of failure.

Biologically, canal isolates frequently expressed virulence determinants (e.g., efaA, gelE) and robust biofilm phenotypes, with associations to radiolucency and retreatment‐related clinical features [21, 30]. Genotyping showed high between‐patient diversity but within‐patient clonality; Brazilian MLST work highlighted recurrent ST397 among endodontic isolates. Multidrug resistance—particularly to macrolides/tetracyclines—was common and, while not dictating intracanal irrigants, mirrors tolerance traits relevant to persistence [16, 25, 27]. These biological traits are also consistent with the classical mechanistic model described by Love [87], who demonstrated that *E. faecalis* can invade dentinal tubules, adhere strongly to type I collagen, and remain viable in the presence of human serum—mechanisms that help explain its persistence in radicular dentine of failed root‐filled teeth. Although Love's study did not appear among the sources retrieved by our search strategy, this was an inherent consequence of our PICOS‐guided search terms, which deliberately emphasised retreatment‐ and intervention‐focused descriptors (e.g., “endodontic retreatment”, “canal retreatment”) rather than broader mechanistic or microbiological terminology. As a result, mechanistic studies not directly linked to retreatment protocols were outside the predefined eligibility criteria. Nevertheless, integrating this foundational evidence strengthens the biological plausibility of the persistence phenomena highlighted by the included studies.

Therapy studies converged on a pragmatic core. Chemo‐mechanical preparation with NaOCl plus EDTA, preferably with activation, and meticulous sealing produced substantial microbial reduction but rarely sterilisation [61, 73, 78, 79]. Two‐visit protocols with intracanal medication improved microbiologic or histologic outcomes in persistent cases, although calcium hydroxide performance was variable and strain/vehicle dependent [60, 63, 77, 80, 82, 83, 85]. Steps that aid removal of legacy materials added antimicrobial dividends—chloroform/xylene outperformed saline during gutta‐percha removal—and light‐based adjuncts (photodynamic therapy, 980‐nm diode) improved microbiology or periapical repair as add‐ons without supplanting conventional protocols [41, 44, 58, 68]. Sealing quality remained decisive: obturation technique and remaining filling length influenced coronal leakage by *E. faecalis* , and ultrasound‐assisted sealer activation improved penetration into isthmuses and superficial dentine with better intradentinal effects [39, 65, 71]. Material‐level findings were heterogeneous—fresh calcium‐silicate sealers often active but attenuating after set; ZOE and some epoxies partially inhibitory; GuttaFlow 2 inactive; and Endox electrofulguration ineffective beyond established irrigants [45, 62, 69, 72, 76, 81]. Matrix disruptors (N‐acetylcysteine), silver nanoparticles and plant‐derived extracts were promising in vitro yet await standardised, clinically anchored trials [48, 49, 50, 51, 52, 57].

In sum, resistant endodontic infections are best addressed by activated NaOCl/EDTA during shaping, consideration of two‐visit strategies with intracanal medication in high‐load or anatomically complex cases, effective solvent‐assisted removal during retreatment, and optimised obturation/sealing including sealer activation. Adjuncts such as PDT/laser, matrix disruptors, nanoparticles and phytochemicals should be framed as add‐ons with encouraging yet still limited clinical corroboration, and future trials should integrate viability‐aware microbiology with patient‐level outcomes to refine protocol selection [34, 36].

## 5 Conclusions

This systematic review confirms that *Enterococcus faecalis* remains a persistent challenge in endodontic failures, with reported prevalence strongly influenced by detection method and sampling strategy. Although diverse interventions—particularly activated NaOCl/EDTA with meticulous sealing, sometimes in two visits—consistently reduce microbial burden, complete elimination is uncommon and material/strain dependent. Adjuncts (e.g., PDT/laser, matrix disruptors, nanoparticles, phytochemicals) show promise mainly as add‐ons rather than stand‐alone solutions. Future work should integrate viability‐aware microbiology with patient‐level outcomes to refine protocols and improve retreatment success.

## Author Contributions

All authors have contributed significantly, and all authors are in agreement with the manuscript.

## Funding

This work was supported by Fundação de Amparo à Pesquisa do Estado de Minas Gerais, APQ‐01203‐23.

## Conflicts of Interest

The authors declare no conflicts of interest.

## Data Availability

The data that support the findings of this study are available from the corresponding author upon reasonable request.
